# Testing the accuracy of a novel digital peak flow meter aligned with a smartphone app compared to a lab spirometer: A pilot work

**DOI:** 10.1177/20552076211005959

**Published:** 2021-05-21

**Authors:** Panagiotis Sakkatos, Andrew Williams

**Affiliations:** 1 Smart Respiratory Products, I-Hub Imperial College London, London, UK; 2 Mid and South Essex NHS Foundation Trust, Broomfield NHS Hospital Allergy Service, Essex, UK

**Keywords:** Smart peak flow, digital peak flow meter, accuracy, asthma

## Abstract

**Background:**

A new digital peak flow meter, known as Smart Peak Flow (SPF), has been developed to monitor asthma patients’ peak expiratory flow (PEF) at home. It is connected wirelessly to any type of smartphone and it is used by asthma patients to self-monitor progress of their clinical condition. Thus evaluation of the SPF’s ability to provide accurate PEF values is essential. The aim of this pilot work was to provide preliminary in-vivo data about the measurement agreement between the SPF and a lab spirometer for a first time.

**Methods:**

PEF measurements were obtained by 9 healthy adults as this pilot work was terminated earlier than it was expected due to COVID-19 restrictions. PEF readings (n=27) were recorded by the comparable devices at the same time during three different expiratory maneuvers performed by each participant. The Bland and Altman plot was used to assess the agreement.

**Results:**

Good agreement between the SPF and the lab spirometer was found with the mean bias being estimated 0.29 L/min. The lower and upper limits of agreement (LOA) were estimated 30.03 L/min and −30.61 L/min respectively.

**Conclusion:**

Due to a small sample size, no firm conclusions can be drawn regarding the SPF’s accuracy. However the current promising results encourage further testing in the future.

## Introduction

International guidelines for asthma self-management suggest the use of peak flow meters for monitoring lung function at home.^1^ Measuring peak expiratory flow (PEF) is an inexpensive and accurate method to evaluate asthma progress.^2^ Nowadays, the use of peak flow meters is highly recommended to monitor asthma while standardised lung function tests, such as spirometry, can be of limited use in clinical settings due to COVID-19 restrictions.^3^ It is therefore essential for peak flow meters to provide accurate measurements following specific criteria as suggested by international guidelines.^4^

Several mechanical peak flow meters have been developed, but validation studies have shown discrepancies between them.^5–7^ These discrepancies have been attributed to methodological differences, such as the use of flow generators instead of human subjects during the developmental phase. A new digital peak flow (Smart Peak Flow-SPF) connected wirelessly to any smartphone, has been developed and it has been found to increase patients’ adherence to self-monitor asthma progress.^8^ This may be because it facilitates the measurement not only of PEF, but also it monitors other important asthma outcomes, such as daily symptoms’ severity, reliever medication usage and the risk for asthma worsening via an Artificial Intelligent algorithm. This can offer a more objective assessment of asthma progress compared to conventional peak flow meters. The SPF is paired with a smartphone app allowing patients to share online their data with their clinicians. This also facilitates remote patient monitoring and avoidance of data fabrication from patients using paper charts. Recent bench test results on the accuracy of nine peak flow meters showed that only the SPF and the Mini-Wright did not fail the accuracy criteria.^7^ However, the accuracy of Smart Peak Flow has not been exclusively tested in human subjects yet. Thus, this observational cross-sectional pilot work aimed to provide preliminary in-vivo data regarding the accuracy of the SPF compared to a lab spirometer.

## Methods

Healthy adults (aged 18 or more) who provided a written consent form, were recruited from offices located at I-Hub Imperial College London. Only 9 healthy volunteers enrolled in this pilot work while COVID-19 restrictions led to an early termination of this pilot work. This pilot work was intended to recruit a minimum of 25 healthy adults and obtain a first data-set to check the SPF’s accuracy prior to the involvement of asthma patients in a future bigger study. A spirometer (Piston PDD-301/sh) was used as the gold-standard monitoring method to compare PEF measurements obtained by the SPF at the sitting position. A single, well-trained researcher was responsible to collect data from the studied subjects who performed three different expiratory maneuvers using a low, medium and maximal effort respectively. Although maximal effort is of clinical significance, obtaining PEF values from healthy individuals during maximal effort could lead to paucity of low PEF values. Therefore, we involved three different expiratory maneuvers which allowed us to check the accuracy of SPF within a wider range of PEF values irrespective of the underlying lung function of the studied subjects. Readings of PEF were recorded by the comparable devices at the same time. The SPF was tested in series connection with the lab spirometer. This testing method has been previously used in the literature as this eliminates any measurement bias potentially induced by intra-patient variability during the different expiratory maneuvers.^9^ It was therefore essential to check whether the performance of the SPF could be disturbed by the spirometer. The SPF was strictly attached directly at the back of the spirometer’s mouthpiece. Uninterrupted airflow passed through both devices at the same time whilst testing it with a calibration syringe.

The SPF’s mouthpiece contains a propeller and a light sensor. The SPF’s PEF was calculated via the rotation of the propeller caused by the air moving into the mouthpiece. This is detected by a light sensor whose signal is translated into an audio signal calculating PEF values on the smartphone app. The latest available version of SPSS was used for statistical analysis. To check the measurement agreement between the comparable methods, a Bland and Altman plot was used to quantify any systematic bias and set limits of agreement (LOA). This was performed by plotting the inter-device differences against spirometry-obtained PEF measurements whilst considering the lab spirometer as a gold standard method.^10^ Acceptable LOA was priori set at 40 L/min as recommended in validation work for peak flow meters.^11^ The Bland and Altman plot was used after achieving normal distribution of the inter-device differences as checked by using Shapiro-Wilk test (Sig. 0.110) and a histogram.

## Results

**Figure 1.**
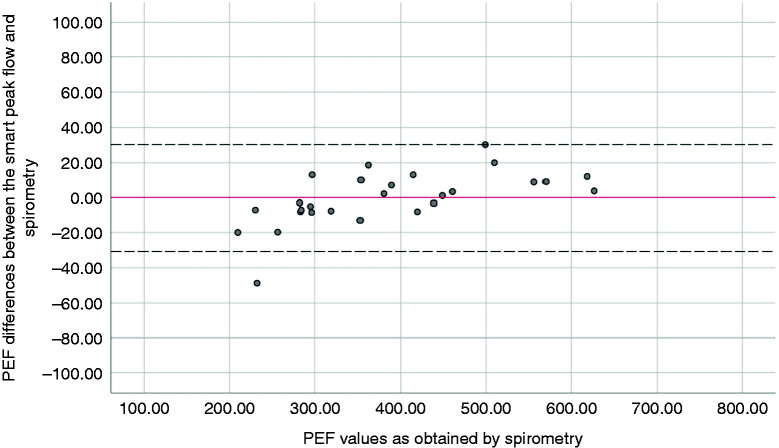
The measurement agreement between the SPF and the spirometer. Red line illustrates the bias (mean difference) between the comparable methods for PEF values. The black dashed lines show upper and lower LOA in each plot.

PEF data (n=27) were analysed from 9 subjects (7 males) whose mean (sd) age and BMI were 41.89 years (13,31) and 24,33 kg/m^2^ (2.99 kg/m^2^) respectively. None of the subjects self-reported a history of chronic lung condition or appeared with fixed airflow obstruction (FEV_1_/FVC<0.70). The Bland and Altman plot showed good agreement between the SPF and the spirometer across a PEF range between 210 L/min and 626.40 L/min (Figure 1). The bias between the SPF and the spirometer was found to be −0.29 L/min with the LOA ranging from 30.03 L/min to −30.61 L/min.

## Discussion

The preliminary data of this pilot work show that the SPF can satisfy the standardised criteria for monitoring devices. Official ATS/ERS statement on PEF measurements recommends that a new peak flow meter should not exceed 20 L/min when compared to a current gold-standard monitoring method.^4^ The averaged bias between the SPF and the lab spirometer was found to be lower than these standardised accuracy criteria. However only 2 out of 27 PEF measurements exceeded the accuracy criteria with only one measurement falling out our expected LOA. Therefore the data presented in this pilot work together with previous published bench test-results show promising results on the SPF’s accuracy.^7^

Since the SPF can acquire more meaningful clinical information about patients’ asthma than conventional peak flow meters, it may facilitate a better method to assess asthma progress and enhance patients’ adherence rates. To date, evidence has showed an increase of patients’ compliance to PEF recordings after using the SPF.^8^ The SPF is able to store a long and detailed history of asthma-related outcomes. These can be shared online with healthcare providers who can instantly review their patients’ asthma condition instead of reviewing paper charts which can be prone to data fabrication.^11^ Considering other drawbacks underlying mechanical peak flow meters, such as induced measurement errors after a prolonged use,^12^ digital peak flow meters, such as the SPF, is likely to maintain better performance over longer periods, but further testing is required.

One of the major limitations of this pilot work is the small sample size. Although 27 PEF measurements were analysed to check the SPF’s accuracy, these measurements came from only 9 healthy volunteers. Thus, no firm conclusions can be drawn regarding the in-vivo accuracy of the SPF. However, considering the promising results of this pilot work and the theoretical advantages of the SPF compared to conventional peak flow meters, it is worth to further test the SPF’s accuracy in a bigger study. This study should involve asthma patients with varying demographic data and disease severity. Finally, the reliability of the SPF should be examined in this future trial as its ability to provide consistent PEF measurements over time is another crucial element of the evaluation process of its monitoring performance.
